# Assessment of Walnut and Quinoa Oil-Based Gelled Emulsion as Partial Animal Fat Replacer in “Longaniza de Pascua” Sausage, a Dry-Cured Meat Product

**DOI:** 10.3390/foods15214001

**Published:** 2026-11-07

**Authors:** Carmen Botella-Martínez, Raquel Lucas-González, José Ángel Pérez-Álvarez, José Manuel Lorenzo, Clemencia Chaves-López, Juana Fernández-López, Manuel Viuda-Martos

**Affiliations:** 1 Research Group in Innovations in Food Products (IPOA), Institute for Agri-Food and Agri-Environmental Research and Innovation, Universidad Miguel Hernández (CIAGRO-UMH), 03312 Orihuela, Spain; c.botella@umh.es (C.B.-M.); raquel.lucasg@umh.es (R.L.-G.); ja.perez@umh.es (J.Á.P.-Á.); j.fernandez@umh.es (J.F.-L.); 2 Centro Tecnológico de la Carne de Galicia, Avd. Galicia No 4, Parque Tecnológico de Galicia, San Cibrao das Viñas, 32900 Ourense, Spain; jmlorenzo@ceteca.net; 3 Área de Tecnología de los Alimentos, Facultad de Ciencias de Ourense, Universidade de Vigo, 32004 Ourense, Spain; 4 Faculty of Bioscience and Technology for Food, Agriculture and Environment, University of Teramo, Via R. Balzarini 1, 64100 Teramo, Italy; cchaveslopez@unite.it

**Keywords:** “Longaniza de Pascua”, vegetable oils, gelled emulsion, animal fat replacement, fatty acid profile

## Abstract

The replacement of animal fat with structured lipid systems offers an approach for improving the nutritional quality of traditional dry-cured meat products. This study evaluated the effect of replacing pork backfat with a quinoa and walnut oil-based gelled emulsion stabilized with pomegranate peel flour (QWP-GE) in “Longaniza de Pascua”-type sausages. A control formulation was compared with sausages in which 25% and 50% of pork backfat was replaced by QWP-GE. The incorporation of QWP-GE substantially modified the fatty acid profile, reducing saturated fatty acids from 41.70 to 32.30 g/100 g fat and increasing polyunsaturated fatty acids from 13.84 to 31.98 g/100 g fat. The contribution of n-3 fatty acids also increased from 0.65 to 3.78 g/100 g fat. These changes were accompanied by improvements in lipid health indices, with the atherogenic and thrombogenic indices decreasing from 0.52 to 0.35 and from 1.32 to 0.72, respectively. QWP-GE incorporation also modified product texture, increasing hardness, cohesiveness, gumminess and chewiness. During ripening, reformulated sausages showed lower weight loss than the control. Lipid oxidation increased following fat replacement, although the TBARS values remained below the indicative acceptability threshold reported for dry-cured meat products. Microbiological quality was not adversely affected. The results indicate that QWP-GE is a promising fat-replacement system for modifying the fatty acid profile and lipid nutritional quality of “Longaniza de Pascua”, although further optimization and sensory validation are required to preserve the quality characteristics of the traditional product.

## 1. Introduction

Animal fat plays a pivotal role in the manufacturing of meat products, contributing significantly to their essential technological, rheological, and sensory attributes [1]. It is crucial for developing characteristic textures, enhancing juiciness, and determining the overall flavor profile, stability, and consumer acceptability of the final product [2]. However, dry-cured meat products are typically high in saturated fatty acids and cholesterol. Excessive and regular dietary intake of saturated fatty acids (SFAs) has been strongly linked to an increased risk of developing cardiovascular diseases, obesity, and other metabolic disorders [3,4,5]. Consequently, contemporary consumers are increasingly demanding healthier food options, posing a major challenge for the meat industry to reformulate traditional products by reducing animal fat content without compromising their distinctive quality characteristics [6].

Improving the nutritional quality of meat products through lipid reformulation often requires reducing animal-derived fat and incorporating oils rich in unsaturated fatty acids from vegetable or marine origins with a healthier lipid profile, rich in monounsaturated and polyunsaturated fatty acids [7]. However, replacing conventional meat fats with liquid oils, with an elevated concentration of unsaturated fatty acids, can alter the physicochemical properties of the lipid phase, which may compromise some of the quality characteristics in the resulting reformulated meat products [8].

In response to this challenge, lipid structuring strategies, particularly the development of gelled emulsions, have emerged as a highly promising approach to replace solid animal fats [9,10]. Gelled emulsions function as efficient fat mimics due to their ability to entrap healthy vegetable or marine oils within a structured polymeric network (often proteins or polysaccharides), effectively simulating the technological behavior and mouthfeel of pork backfat [11,12]. Furthermore, these systems allow for the incorporation of bioactive ingredients, which can provide antioxidant properties and dietary fiber [13].

While numerous studies have explored the application of gelled emulsions in fresh meat products such as burgers or patties [14,15] and cooked meat systems including frankfurters, bologna, or pâtés, among others [16,17,18], research regarding their utilization in raw dry-cured meat products remains considerably scarce [19,20]. Dry-cured matrices undergo complex ripening, drying, and lipolysis processes where fat stabilization is critical, making the study of fat replacers in these products both novel and challenging [21].

In the case of fresh and cooked meat products where gelled emulsions have been successfully applied, the lipid profile of the reformulated meat products is impacted depending on the fatty acid profile of the oils and the level of replacement. In some cases, even 100% of the animal fat was successfully substituted with the gelled emulsion [13,14,16], while in other cases only partial replacements were achieved [15,17]. In all cases, significant reductions in the proportion of SFAs (up to 63%) and increases in unsaturated fatty acids (UFAs), both mono- (MUFAs) and poly- (PUFAs) unsaturated fatty acids depending on the vegetable oil used, were achieved compared to the control products.

Dry-cured meat products represent an important category of traditional meat products in Spain, with an estimated annual per capita consumption of approximately 8.4 kg. This consumption level highlights the relevance of these products in the Spanish diet. Among traditional dry-cured meats, “Longaniza de Pascua” holds a prominent position, particularly within the gastronomy of the Spanish Mediterranean region. It is a traditional Spanish dry-cured sausage made from lean pork and pork backfat, all finely minced and mixed with species, stuffed into a narrow casing (about 15–22 mm diameter) and dried at low temperature for around 10–15 days [22]. During this process, a rapid water activity decrease occurs, which constitutes the first defense against the proliferation of pathogens. Under these conditions, a short spontaneous fermentation occurs, reaching a final pH above 5.3 [23], which is highly appreciated by consumers who reject an excessive acidic taste [24]. These specific process conditions result in sausages more tender than other types of cured sausages, but with a firm, meaty texture, pinkish color and fairly natural flavor, with distinct hints of spices. This sausage is highly appreciated for its unique sensory profile, short ripening period, and deep-rooted cultural heritage. Given its socioeconomic relevance and specific manufacturing traditions, “Longaniza de Pascua”-type sausage is recognized under specific quality designations and traditional product registries that protect its identity and manufacturing standards. Preserving the traditional essence of this product while adapting it to modern nutritional recommendations through the incorporation of structured healthy oils represents a significant advancement in the development of functional and healthier traditional meat products.

In this context, the objective of this work was to investigate the effects of partially replacing pork backfat (25% and 50%) with a quinoa and walnut oil-based gelled emulsion, supplemented with pomegranate peel powder, on the physicochemical, nutritional, textural, and microbiological quality and the lipid oxidation stability of “Longaniza de Pascua” dry-cured sausages. Quinoa and walnut oils were selected as sources of unsaturated fatty acids to improve the lipid profile due to both oils being particularly rich in linoleic and oleic acids, contributing to the nutritional value of the reformulated product. However, the high degree of unsaturation may increase susceptibility to lipid oxidation, particularly during the drying and ripening of dry-cured sausages. Pomegranate peel powder was therefore incorporated as a source of phenolic compounds with antioxidant properties, potentially limiting oxidative deterioration during processing and storage. In addition to its potential antioxidant activity, the powder may influence the physical properties of the emulsion through interactions between its constituents and the gelled matrix.

## 2. Materials and Methods

### 2.1. Materials

To prepare the gelled emulsions, the following components were used: quinoa seed oil (50.80% C18:2*n-6,9;* 29.36% C18:1*n-9;* 8.85% C16:0; and 5.58% C18:3*n-3,6,9*) acquired from Bela Vizago (Murcia, Spain) and walnut oil (61.37% of C18:2*n-6,9*; 14.41% C18:1*n-9*; 14.12% C18:3*n-3,6,9*; and 6.62% C16:0) obtained from Cooks&Co (Isleworth, UK); lyophilized pomegranate peel flour obtained from pomegranate fruits (*Punica granatum*) var. mollar de Elche that were harvested from the experimental field station of Miguel Hernandez University in Orihuela, Spain, at the commercial ripening stage. Gelatin from animal origin (pork) with 240 bloom was supplied by Mondelez S.L. (Madrid, Spain). The meat ingredients (pork backfat and pork meat) were acquired from a local butcher in Orihuela (Spain), while the spices and additives were supplied by Suministros River S.L.U. (Orihuela, Spain).

### 2.2. Gelled Emulsion Elaboration

The gelled emulsion (QWP-GE) was elaborated following the methodology described by Botella-Martínez [25] using the following ingredients: water (47.5%), walnut oil (20.0%), quinoa seed oil (20.0%), gelatin (7.0%), pomegranate peel flour (5.0%), and sodium chloride (0.5%). Briefly, gelatin was first dispersed in 80% of the total water and heated to 65 °C under mixing until complete dissolution. The remaining 20% of the water was used to prepare a NaCl solution. Both aqueous phases were subsequently combined and incorporated into the pomegranate peel flour, followed by homogenization using a blender. Once a uniform dispersion was obtained, walnut and quinoa seed oils were progressively incorporated under continuous blending. The resulting QWP-GE was subsequently refrigerated and maintained at 4 °C until further use. The developed QWP-GE had the following properties: the pH value was 4.31, while the emulsion stability assessed using the methodology reported by Pintado et al. [26] reached 98.97%; regarding color properties, L*, a*, b*, C*, and h* values were 71.62, (−0.09), 21.55, 21.57, and 90.43, respectively.

### 2.3. “Longaniza de Pascua”-Type Sausage Elaboration

“Longaniza de Pascua”-type sausages were elaborated in the pilot plant of CIAGRO-UMH (Universidad Miguel Hernández). Pork lean meat (68.65% moisture, 26.92% protein, 3.64% lipids, and 0.79% ash) and pork backfat (11.68% moisture, 3.58% proteins, 84.29% lipids, and 0.45% ash) were used for the elaboration of three formulations of “Longaniza de Pascua”-type sausages. A control formulation, with a regular fat content, was elaborated with pork lean meat (53.58 g/100 g), pork backfat (35.73 g/100 g), ice (8.93 g/100 g), NaCl (1.34 g/100 g), and the remaining additives and spices per 100 g of meat batter, as detailed in Table 1. To determine the effect of animal fat replacement, two preparations were established (Table 1), with 25% (ES25) and 50% (ES50) pork backfat substitution by QWP-GE.

**Figure 1:**
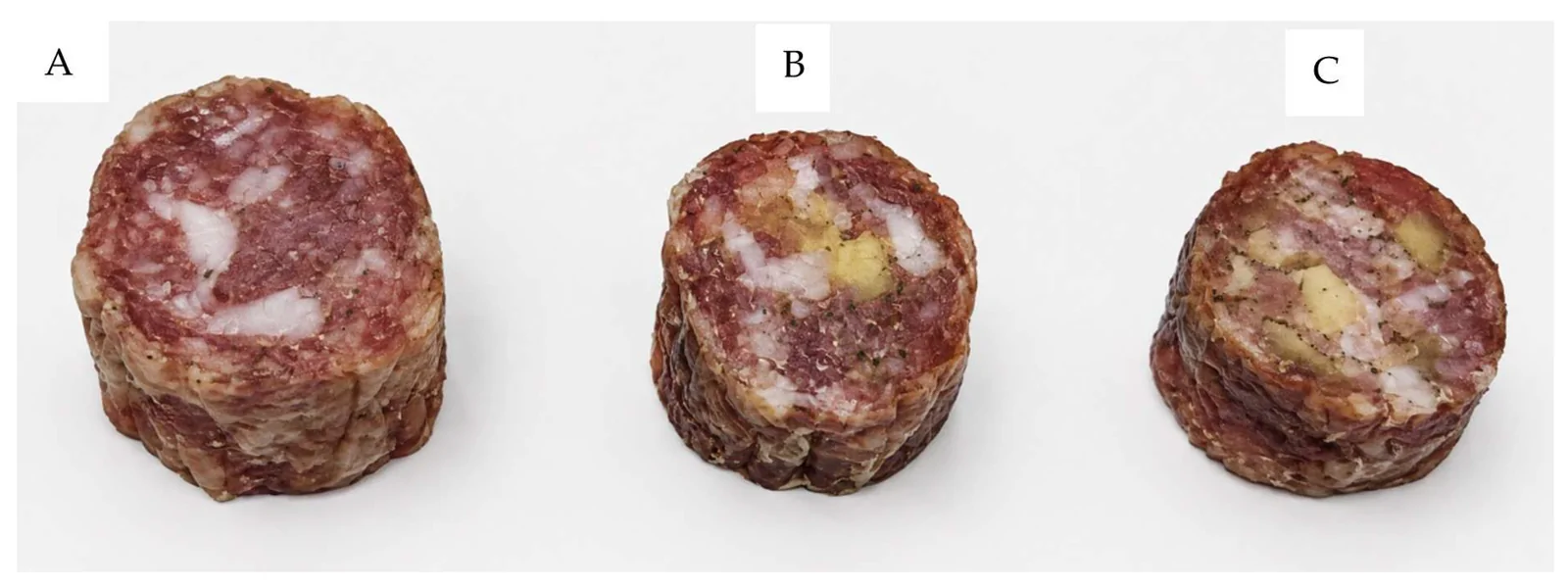
(**A**): Control sample produced with conventional fat content; (**B**): S25: reformulated samples in which 25% of pork backfat was replaced by oil-in-water emulsion prepared with quinoa and walnut oils and stabilized using pomegranate peel flour; (**C**): S50: reformulated samples in which 50% of pork backfat was replaced by oil-in-water emulsion prepared with quinoa and walnut oils and stabilized using pomegranate peel flour.

The preparation of “Longaniza de Pascua” sausages began by mincing the lean pork and pork backfat separately through an 8 mm plate. The minced materials were subsequently combined with the remaining ingredients in a bowl mixer equipped with a spiral hook and mixed for approximately 8 min, until a uniform and cohesive meat batter was obtained. The resulting mixture was refrigerated at 4 °C for 1 h to allow sufficient compaction before stuffing. Subsequently, the chilled meat batter was introduced into an Talsa F14s piston stuffer (Talsa, Almazora, Spain) and filled into 30 mm diameter artificial collagen casings (Fibran-Pack, Fibran Group, Barcelona, Spain), which had previously been hydrated in water at 4 °C. The filled casings were manually portioned into links of approximately 15 cm in length. Finally, the sausages were rinsed to eliminate any residual material adhering to the outer surface of the casings. The sausages were dry-cured in a controlled chamber, Infrico AN501SC (Infrico S.L., Lucena, Spain), where the temperature and relative humidity were set at 16 ± 1 °C and 78 ± 2%, respectively. During the first 48 h, the air velocity ranged from 0.3 to 0.5 m/s. Thereafter, the air velocity was maintained at 0.2 m/s until the end of the curing process. At the end of the dry-curing period, sausages are considered “ready-to-eat”, meaning that there is no need to apply thermal treatment before consuming them (Figure 1). To monitor the dry-curing process, weight loss was determined on days 1, 2, 3, 4, 5, 6, 7 and 8, while the water activity was assessed on days 0, 2, 4, 6 and 8.

### 2.4. Chemical Composition

“Longaniza de Pascua”-type sausages were assessed for their moisture, protein, fat and ash contents following the methodology of the Association of Official Analytical Chemistry (AOAC) [27]. The values were expressed as g/100 g. The residual nitrite content, expressed as mg NaNO_2_/kg sample, was determined following the ISO/DIS 2918.26 guidelines [28].

To determine the fatty acid profile, the lipid content was extracted from “Longaniza de Pascua”-type sausage according to the method describe by Folch et al. [29]. The fatty acids were methylated following the methodology reported by AOAC [27] to obtain the corresponding fatty acid methyl esters (FAMEs). FAMEs were analyzed using a gas chromatograph (HP-6890) equipped with an FID detector and a SupraWax 280 column (30 m × 0.25 mm i.d., 0.25 μm film thickness; Teknokroma, Barcelona, Spain) following the chromatographic conditions defined by Cîrstea et al. [30] with some modifications. Individual fatty acids were identified by comparing their retention times with those of a certified standard mixture (Supelco 37 Component FAME Mix, Bellefonte, PA, USA). The relative abundance of each fatty acid was subsequently calculated from the corresponding chromatographic peak areas and expressed as grams of fatty acid per 100 g of total fat.

### 2.5. Nutritional Parameters, and Health Indices Analysis

The nutritional indices refer to the sum of total saturated fatty acids (SFAs), monounsaturated fatty acids (MUFAs), polyunsaturated fatty acids (PUFAs)—the amount of omega 3 (n-3) and omega 6 (n-6) fatty acids present in “Longaniza de Pascua”-type sausage. The thrombogenicity index (TI) and the atherogenicity index (AI) were calculated using equations 1 and 2, respectively, as proposed by Ulbricht and Southgate [31]. The hypocholesterolemic/hypercholesterolemic ratio (h/H) was determined as reported by Chen and Liu [32], using equation 3. The n-3/n-6 and SFA/UFA ratios were also calculated:

(1) $$\mathrm{AI}=\;\frac{C12:0+\left(4\times C14:0\right)+C16:0}{\sum \mathrm{MUFA}+\sum n6+\sum n3}$$

(2) $$\mathrm{TI}=\frac{C14:0+C16:0+C18:0}{(0.5\;\times \sum \mathrm{MUFA})+(0.5\times \sum \mathrm{PUFA n}6)+(3\times \sum \mathrm{PUFA n}3)+\frac{\sum n3}{\sum n6}}$$

(3) hH=C18:1 n−9+ C18:1 n−7+∑PUFA(C14:0+ C16:0)

### 2.6. Physico-Chemical Analysis

The pH of the “Longaniza de Pascua”-type sausages was measured by direct insertion of a puncture electrode Testo206-pH2 (Testo, Titisee-Neustadt, Germany) coupled to a Crison-GLP21 pH meter with automatic temperature compensation (Crison Instruments S.A., Barcelona, Spain). Prior to each series of measurements, the equipment was standardized against buffer solutions at pH 4.01, 7.00, and 10.00.

Water activity (aw) was measured at 25 °C with a LabSwift-aw hygrometer (Novasina AG, Lachen, Switzerland).

Instrumental color measurements were performed using a CM-700 spectrophotometer (Konica Minolta, Tokyo, Japan) using the illuminant D_65_ with a standard observer of 10°. Measurements were conducted in SCI mode using an 11 mm illumination aperture and an 8 mm measurement aperture. A spectrally pure glass plate (Minolta CR-A51/1829-752) was placed between the sample and the instrument during analysis. The color parameters obtained in the CIELAB system included lightness (L*), the red–green coordinate (a*), and the yellow–blue coordinate (b*). This measurement was carried out following the procedure of Cassens et al. [33]. Hue angle (h) and chroma (C*) were subsequently derived from these coordinates according to Equations (4) and (5), respectively. In addition, the overall color variation (ΔE) of each reformulated “Longaniza de Pascua” sausage was calculated relative to the control formulation (C) according to Equation (6).

(4) $$C*=\sqrt[{2}]{{{{{(a}^{*})}^{2}+(b}^{*})}^{2}}$$

(5) $$h=\mathrm{arctg}\frac{b^{*}}{a^{*}}$$

(6) $${\Delta E}^{*}=\sqrt[{2}]{{\left(L_{I}^{*}-L_{C}^{*}\right)}^{2}+{\left(a_{I}^{*}-a_{C}^{*}\right)}^{2}+{\left(b_{I}^{*}-b_{C}^{*}\right)}^{2}}$$

Texture parameters were assessed by means of a TA-XT2i texture analyzer (Stable Micro Systems, Surrey, UK). Samples were prepared as cubes of approximately 1 cm^3^ and analyzed under a double-compression test. A deformation level of 75% was applied at a crosshead speed of 60 mm/min, using a trigger force of 10 g and allowing a 5 s interval between successive compressions to enable structural recovery. Measurements were performed at 18 ± 2 °C. The force–time deformation profiles generated during the test were used to assess hardness (N), springiness (mm), cohesiveness, gumminess (N), and chewiness (N·mm), following the methodology described by Claus [34].

### 2.7. Lipid Oxidation

Lipid oxidation in “Longaniza de Pascua”-type sausage was determined by measuring thiobarbituric acid-reactive substances (TBARs), following the MDA extraction procedure proposed by Rosmini et al. [35]. The values obtained were reported as milligrams of malondialdehyde (MDA) per kilogram of sample.

### 2.8. Microbiological Analysis

Microbiological counts were determined at the end of dry-curing period (day 8). For this, 10 g aliquot of each “Longaniza de Pascua”-type sausages was aseptically attained and then homogenized with 90 mL of peptone water (Oxoid, UK) at 1%, in a Stomacher BagMixer 400CC (Interscience, Saint-Nom-la-Bretèche, France) for 2 min.

For microbiological counts, sample aliquots were subjected to serial ten-fold dilutions in sterile 0.85% saline. *Lactobacillus* spp. counts were quantified by spreading the appropriate dilutions onto MRS agar, followed by anaerobic incubation at 37 °C for 72 h in an Anaerocult A system (Merck, Darmstadt, Germany). The populations of total aerobic mesophilic microorganisms, *Enterobacteriaceae*, molds, and yeasts were assessed using Petrifilm plates (3M, Madrid, Spain). Incubation was performed at 37 °C for 24 h for total aerobic mesophilic bacteria and Enterobacteriaceae, whereas plates used for mold and yeast enumeration were maintained at 26 °C for 96 h. Following incubation, plates yielding 30–300 colonies were selected for manual counting. Microbial loads were reported as log10 CFU/g of sample.

### 2.9. Statistical Assay

The production of “Longaniza de Pascua”-type sausages was carried out in three independent manufacturing batches, each prepared on a separate processing day. All analytical determinations were performed in triplicate for every batch. The mean of the three analytical determinations obtained for each independent production batch was used as one experimental observation. Thus, the independent production batch was considered the experimental unit, and the statistical sample size was *n* = 3 per formulation. The results obtained were presented as mean values accompanied by their corresponding standard deviations in the respective tables and figures. Statistical analyses were conducted using IBM SPSS Statistics software (version 26; IBM Corp., Chicago, IL, USA). Significant differences among treatments were assessed by one-way analysis of variance (ANOVA) at a 95% confidence level (*p* < 0.05). When significant effects were spotted, Tukey’s post hoc test was carried out to detect differences between samples.

## 3. Results

### 3.1. Dry-Cured Process

**Figure 2:**
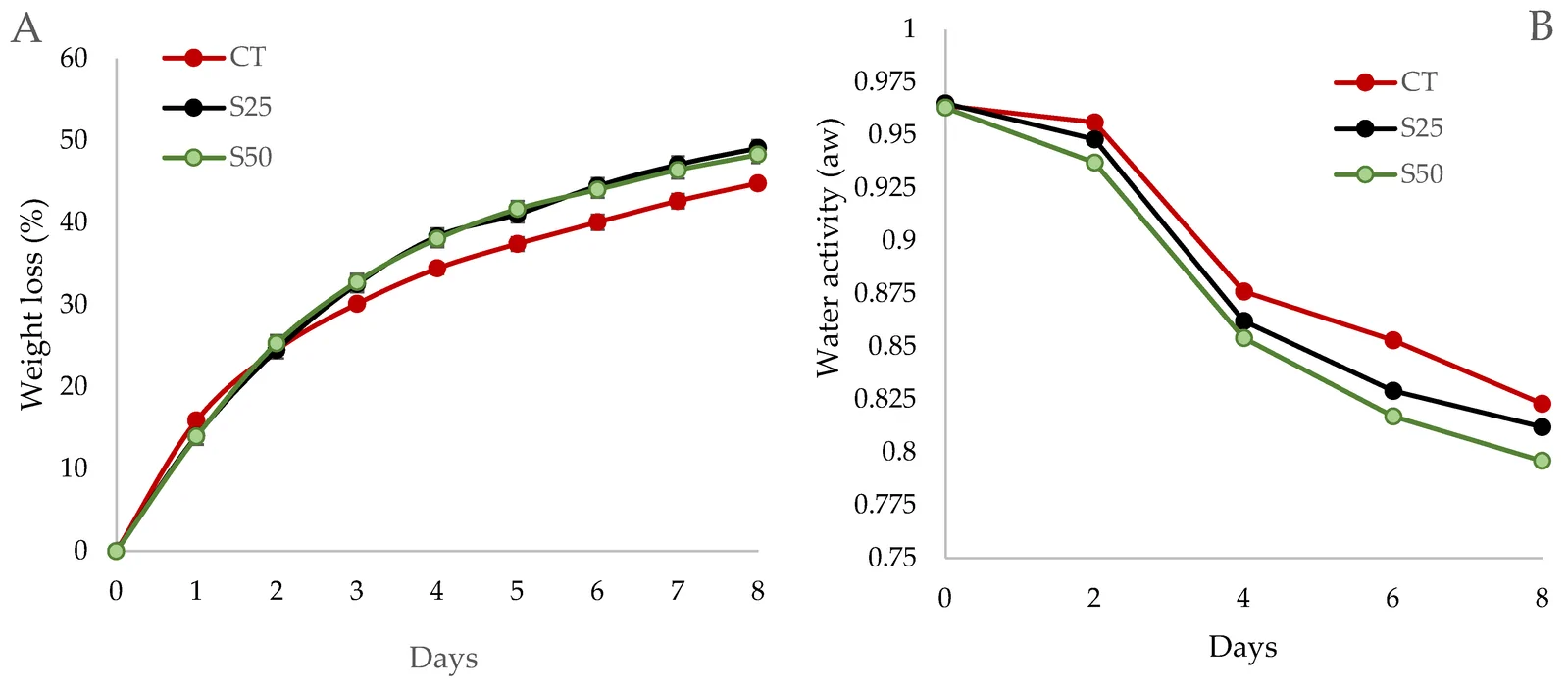
Evolution of (**A**) weight loss (%) and (**B**) water activity (a_w_) throughout the ripening process of control “Longaniza de Pascua”-type sausage (CT) and “Longaniza de Pascua”-type sausage reformulated with QWP-GE used as a partial animal fat replacer at 25% (S25) and 50% (S50). QWP-GE: oil-in-water emulsion prepared with quinoa and walnut oils and stabilized using pomegranate peel flour. CT: control sample produced with conventional fat content; S25: reformulated samples in which 25% of pork backfat was replaced by QWP-GE; S50: reformulated samples in which 50% of pork backfat was replaced by QWP-GE.

Weight loss is considered one of the most critical parameters during the ripening process of dry-fermented sausages, since it directly influences product stability, texture development, and quality characteristics [36]. Figure 2 shows the evolution of weight loss and water activity (a_w_) throughout the ripening process of “Longaniza de Pascua”-type sausages elaborated with different levels of animal fat replacement and control. As expected, all formulations exhibited a progressive reduction in weight throughout the ripening period, reflecting the continuous migration and evaporation of water from the product matrix. However, differences were observed among formulations depending on the level of fat replacement.

The control sample showed the greatest weight loss during the ripening process (55.21%), whereas the reformulated samples exhibited a lower reduction in weight (50.92% and 51.72%, for S25 and S50, respectively). These findings are contradictory to those reported by Siachou et al. [36], who indicated that dry-fermented sausages in which pork fat was replaced with an olive oil-based bigel exhibited greater weight loss than the control samples, and Pintado and Cofrades [22], who reported that fuet-type sausages reformulated with gelled emulsions or oleogels prepared using olive and chia oils exhibited greater weight loss than those formulated exclusively with animal fat. On the other hand, Franco et al. [19] reported that the moisture content in fermented sausages where the pork backfat was replaced by linseed oil oleogels was lower than full-fat sausage. Similar behavior was reported by Cittadini et al. [37] in dry-fermented foal sausages, where the animal fat was partially replaced by a mixture of tigernut and sesame oils.

These differences in the scientific literature might be explained by moisture loss during the ripening process being largely affected by the environmental conditions established within the fermentation chamber, particularly temperature, relative humidity, air speed, and circulation rate, as well as time [38]. Furthermore, the chemical composition and structural organization of the fat replacer may also influence the dehydration kinetics and, consequently, the overall drying behavior of the sausages [39].

In reference to water activity, at the beginning of the ripening process, all samples exhibited high aw values, between 0.963 and 0.965, without statistical differences between samples (*p* > 0.05), which are close to those typically observed in fresh fermented meat products. As ripening progressed, water activity gradually decreased in all formulations as a consequence of moisture loss.

At the end of the ripening process, S50 showed the lowest (*p* < 0.05) value (0.796), while the control sample had the highest (*p* < 0.05). The values obtained for this parameter agree with those reported for this type of dry-cured meat product [22] that guarantee microbiological stability and maintain characteristic texture.

### 3.2. Chemical Composition

The partial replacement of pork backfat with QWP-GE gelled emulsion significantly influenced the chemical composition (expressed as g/100 g dry basis; Supplementary Table S1 expresses the results in g/100 g fresh-weight basis) of the “Longaniza de Pascua”-type sausages, as shown in Table 2. Significant differences (*p* < 0.05) were observed between the mean protein, fat, and ash contents of the reformulated products and those of the control.

With regard to the moisture content, the control sample showed a final content of 31.66 g/100 g without differences (*p* > 0.05) with the S25 sausages, whereas the S50 sausages had the lowest moisture content (*p* < 0.05). The values obtained for control and reformulated sausages were in agreement with those reported by several authors for this type of dry-cured sausage [39,40].

Protein content, expressed on a dry basis (d.b.), was significantly higher (*p* < 0.05) in the reformulated sausages than in the control, increasing from 33.70 g/100 g d.b. in the control to 37.18 and 38.19 g/100 g d.b. in S25 and S50, respectively. This trend is consistent with the findings of Siachou et al. [36], who indicated that dry-fermented sausages reformulated with an olive oil-based bigel had higher protein content than the control. This increase may be explained by the pomegranate peel flour itself containing small amounts of proteins, which may contribute marginally to the overall protein fraction. Additionally, another source that might increase the protein content in reformulated sausages is the use of pork gelatin in the elaboration of the gelled emulsion, which contributes to the total nitrogen content. However, it is important to highlight that gelatin and meat proteins have different nitrogen-to-protein conversion factors (5.55 and 6.25, respectively), which should be considered.

Regarding ash content, the replacement of fat with the QWP-GE emulsion had a significant effect (*p* < 0.05) on this parameter. The control sample exhibited the lowest values (*p* < 0.05), whereas no differences (*p* > 0.05) were observed among the reformulated samples. A similar trend has also been reported in several studies on dry-cured sausages in which animal fat was partially replaced with gelled emulsions [37,40]. The higher ash content of the reformulated samples may partly reflect the mineral contribution of the pomegranate peel flour, which contains minerals such as potassium and calcium and other micronutrients [41]. In addition, the incorporation of the aqueous and gelatin-containing phases changes the proportions of the formulation components and may influence the relative concentration of non-combustible constituents in the final product.

As expected, the fat content (Table 2) was lower (*p* < 0.05) than control sample due to the use of QWP-GE as a fat replacer. Thus, S25 and S50 achieved a reduction of 10.96% and 20.36% compared with the control sample. This reduction was expected, given that the fat content of the pork backfat was 84.29%, whereas the oil content in the QWP-GE was 40%.

Consequently, replacing pork backfat with QWP-GE reduced the amount of lipid introduced into the formulation. However, this effect should be interpreted in the context of the overall composition of the gelled emulsion, which also contains water, gelatin, and pomegranate peel flour. These components contribute to the non-lipid fraction of the formulation and therefore also influence the final proximate composition. These reductions were in concordance with other works on dry-cured sausages in which the animal fat was replace by gelled emulsions [36,39,42].

In reference to residual nitrite content (Table 2), the control sample showed the highest value, followed by S25, without differences (*p* > 0.05) among them. On the other hand, S50 had the lowest (*p* < 0.05) residual nitrite levels. The lower residual nitrite levels in the reformulated samples may be associated with the bioactive compounds present in the pomegranate peel, which could enhance nitrite reactions and thereby reduce the residual nitrite concentration [43].

The effect of partial replacement of pork backfat by QWP-GE gelled emulsion on the fatty acid profile (g/100 g of fat) of “Longaniza de Pascua”-type sausages is showed in Table 3. As expected, replacing animal fat with QWP-GE significantly affected (*p* < 0.05) the fatty acid composition of “Longaniza de Pascua”. In all formulations, monounsaturated fatty acids (MUFAs) were the predominant lipid fraction, followed by saturated fatty acids (SFAs) and polyunsaturated fatty acids (PUFAs). Similar fatty acid distributions have been reported in dry-fermented foal sausages [37] and dry-fermented deer sausages formulated with gelled emulsions as animal fat replacers [39].

Regarding SFAs, replacing pork backfat with QWP-GE reduced total SFA content by 10.14% and 22.54% in S25 and S50, respectively, compared with the control. With the exception of C12:0, C17:0, and C23:0, which were minor fatty acids (<0.55% of total fatty acids), all individual SFAs decreased significantly (*p* < 0.05) following fat replacement. In contrast, PUFA content increased by 59.89% and 131.07% in S25 and S50, respectively, relative to the control. This increase was mainly attributable to the higher concentrations of linoleic acid (C18:2n-6,9) and α-linolenic acid (C18:3n-3,6,9). Specifically, linoleic acid increased by 57.78% and 126.65% in S25 and S50, respectively, compared with the control. Likewise, α-linolenic acid increased from 0.65 g/100 g fat in the control to 2.00 and 3.66 g/100 g fat in S25 and S50, respectively.

These findings are consistent with those reported for fresh, cooked, and dry-cured meat products in which animal fat was replaced with gelled emulsions formulated using vegetable oils rich in polyunsaturated fatty acids [14,16,20,37].

Partial replacement of pork backfat with QWP-GE significantly increased (*p* < 0.05) the total contents of both ω-3 and ω-6 fatty acids in a dose-dependent manner (Table 3). Total ω-3 fatty acids increased from 0.65 g/100 g fat in the control to 2.07 and 3.78 g/100 g fat in S25 and S50, respectively, whereas total ω-6 fatty acids increased from 12.20 (control sample) to 19.16 and 27.47 g/100 g fat in S25 and S50, respectively. These changes are consistent with the fatty acid profile of walnut and quinoa oils, which are naturally rich in essential polyunsaturated fatty acids.

### 3.3. Health Indices Analysis

The replacement of pork backfat with QWP-GE resulted in an improvement in the theoretical nutritional quality of the fatty acid profile of “Longaniza de Pascua”-type sausage, as indicated by the calculated lipid nutritional indices (Table 4). The atherogenic index (AI) decreased significantly (*p* < 0.05) from 0.52 in the control to 0.44 and 0.35 in S25 and S50, respectively. A similar trend was observed for the thrombogenic index (TI), which was reduced from 1.32 in the control to 1.00 and 0.72 (*p* < 0.05) following the incorporation of 25% and 50% QWP-GE, respectively.

These reductions are consistent with the lower proportion of saturated fatty acids and the concurrent increase in unsaturated fatty acids associated with the replacement of pork backfat with the walnut- and quinoa-oil-based gelled emulsion. Lower AI and TI values are generally considered indicative of a more favorable fatty acid profile according to these theoretical indices [44]. However, these indices reflect the composition of dietary fatty acids and should not be interpreted as direct evidence of cardiovascular benefits or a reduced risk of cardiovascular disease in consumers. Such health outcomes cannot be established from fatty acid composition alone and would require additional evidence beyond the scope of the present study. The hypocholesterolemic/hypercholesterolemic fatty acid ratio (h/H) increased significantly (*p* < 0.05) from 2.14 in the control sample to 2.57 and 3.20 in S25 and S50, respectively. This index reflects the balance between fatty acids that contribute to lowering plasma cholesterol concentrations and those associated with increased cholesterol levels. Consequently, the progressive increase in the h/H ratio indicates a more favorable fatty acid composition in reformulated samples [45]. The values obtained in S50 are particularly noteworthy, representing an increase of almost 50% relative to the control formulation.

This improvement is consistent with the higher concentrations of linoleic and α-linolenic acids supplied by vegetable oils, together with the reduction in the main hypercholesterolemic saturated fatty acids. The values obtained for TI, AI and h/H indices are consistent with previous studies reported by other authors [35,46] who applied gelled emulsions formulated using vegetable oils rich in polyunsaturated fatty acids as fat replacers in meat products.

The saturated-to-unsaturated fatty acid ratio (SFA/UFA) also decreased significantly from 0.71 in the control to 0.60 and 0.48 in S25 and S50, respectively. A lower SFA/UFA ratio is generally considered indicative of improved nutritional quality because it reflects a greater proportion of health-promoting unsaturated fatty acids [47]. The incorporation of QWP-GE also produced a substantial increase in the n-3/n-6 ratio; thus, S25 and S50 samples showed a rise of 120 and 180%, respectively, with respect to control samples. The observed increase showed that replacing pork backfat with a vegetable oil-based structured lipid system substantially enhances the contribution of ω-3 fatty acids, confirming the results obtained by several authors [7,15]. This improvement is mainly explained by the high α-linolenic acid content naturally present in walnut and quinoa oils.

### 3.4. Physico-Chemical Properties

The pH values as well as the color properties of control “Longaniza de Pascua”-type sausage and “Longaniza de Pascua”-type sausage reformulated with the QWP-GE gelled emulsion used as a partial animal fat replacer are shown in the Table 5. The pH values were affected significantly (*p* < 0.05) by the pork backfat substitution. Thus, the pork backfat replacement by QWP-GE gelled emulsion decreased the pH values in a concentration-dependent manner. In the scientific literature, there are contrary findings in reference to the effect of substituting animal fat with gelled emulsions elaborated with vegetable oils on the pH values of dry-cured meat products. Thus, the results obtained in this work agree with those reported by Pintado and Cofrades [20], who reported that the use of emulsion gel as an animal fat replacer in “fuet” resulted in the pH value decreasing from 5.50 to 5.20; on the other hand, Siachou et al. [36] and Zampouni et al. [48] mentioned that the pH values of fermented sausages were not affected by fat replacement with bigels or olive oil structured with monoglycerides as compared to control sausages.

Color is one of the visual attributes that may contribute to consumers’ perceptions of the quality and acceptability of dry-cured sausages. In particular, noticeable color defects, such as greenish discoloration or an abnormally faded appearance, may negatively influence product perception. However, the importance of color may depend on the characteristics and presentation of the product as well as on other sensory attributes that contribute to overall consumer acceptance [49].

The color properties obtained for the control “Longaniza de Pascua”-type sausage and “Longaniza de Pascua”-type sausages reformulated with QWP-GE gelled emulsion used as a partial animal fat replacer are shown in Table 5. The use of QWP-GE gelled emulsion as a partial animal fat replacer led to significant changes (*p* < 0.05) in all color coordinates (except the lightness) as well as the psychophysics properties of reformulated “Longaniza de Pascua”-type sausages. Regarding lightness (L*), the substitution of pork backfat with the QWP-GE gelled emulsion had no significant effect on this parameter. Therefore, no statistically significant differences (*p* > 0.05) were observed among CT, S25, and S50. These results were contradictory with those reported by several authors [37,39] who mentioned that the L* values of dry-cured sausages where part of the animal fat was replace by gelled emulsions were lower than full-fat sausages. Indeed, Szydłowska et al. [50] reported that fat content is a crucial factor influencing L* values, with higher fat levels in sausages generally resulting in increased lightness. However, it should be borne in mind that the lightness of dry-fermented sausages is influenced by several factors, including, water content, meat pigments, matrix structure, and the physicochemical changes occurring during ripening [51]. For red-green coordinate (a*), the substitution of animal fat with QWP-GE reduced the values for this coordinate, with statistical differences (*p* < 0.05) with control sample (Table 5). However, no statistically significant differences (*p* > 0.05) were observed among S25 and S50. In the case of the yellow-blue coordinate (b*), the replacement of pork backfat with QWP-GE increased the values for this coordinate, with statistical differences (*p* < 0.05) with the control sample (Table 5). In the scientific literature, there are contradictory results for coordinates a* and b* when gelled emulsions elaborated with vegetable or fish oils were used as animal fat replacers in dry-cured sausages [20,39,40]. The differences reported across studies can be related to the inherent color of the emulsifiers, the type of oil selected for gel preparation, the oil concentration within the emulsion, and the composition of the meat formulation. Variations in these factors might modify the optical properties of the resulting matrix and account for the contrasting color responses described in the literature.

This behavior was also found for the psychophysics properties Chroma (C*) and hue (h). The replacement of pork backfat with QWP-GE supplemented with purple sweet potato peel extract, at both contents, provokes a significant increase (*p* < 0.05) in the values of these parameters for S25 and S50 with respect to the control sample. The significance of these color variations in the reformulated “Longaniza de Pascua”-type sausages is also reflected in the color differences (ΔE*) between the sausages supplemented with QWP-GE and the control samples (Table 5). In this way, both S25 and S50 “Longaniza de Pascua”-type sausages had color differences higher than 3.5, which correspond to clear differences in the color of the samples under comparison following the criteria of Barroca et al. [52], indicating an instrumentally detectable color difference whose effect on consumer acceptance requires sensory verification.

In the texture profile analysis (TPA) of dry-cured sausages such as “Longaniza de pascua”, hardness, cohesiveness, springiness, gumminess and chewiness are the principal mechanical parameters that determine the textural characteristics. The TPA (Table 5) assay revealed that the partial replacement of animal fat with QWP-GE modified several (*p* < 0.05) textural attributes of the “Longaniza de pascua”-type sausage, particularly hardness, cohesiveness, gumminess, and chewiness, whereas springiness remained unaffected (*p* > 0.05). For hardness, the control sample showed the lowest values (*p* < 0.05), whereas the incorporation of QWP-GE increased hardness progressively, with no significant differences between S25 and S50 (*p* > 0.05). This increase in the S25 and S50 hardness may be due to their lower moisture content. According to Szydłowska et al. [50], there is a negative correlation between hardness and moisture content in dry fermented meat products. Thus, water acts as a plasticizing component within the protein matrix, reducing intermolecular interactions and facilitating matrix deformation. Conversely, lower water availability generally results in a more concentrated and compact solid matrix, which can increase mechanical resistance [53]. The results obtained were consistent with those reported by Pintado and Cofrades [20] in “fuet” and by Cittadini et al. [37] in dry-fermented foal sausages, in which part of the animal fat was replaced with a gelled emulsion prepared using vegetable oils.

In reference to springiness (Table 5), no statistical differences (*p* > 0.05) were found between CT, S25 and S50. The absence of significant differences indicates that the use of QWP-GE as fat replacer did not substantially modify the ability of the sausages to recover their original shape following compression. With regard to cohesiveness (Table 5), as occurs with hardness, the CT sample showed the lowest value (*p* < 0.05), while no significant statistical differences (*p* > 0.05) were found between S25 and S50 samples. This increase in cohesiveness may be associated with improved structural integration of the structured lipid phase within the meat matrix. Gumminess (Table 5) exhibited a similar trend to that observed for cohesiveness. The S25 and S50 samples showed the highest values (*p* < 0.05) for this parameter, without differences (*p* > 0.05) between them. The increase for this parameter as observed in these samples may reflect the greater structural organization and internal resistance of the reformulated meat matrix. The gelled emulsion may have promoted stronger interactions between the lipid phase and the surrounding protein network, thereby generating a more cohesive structure that was more resistant to deformation [54]. Regarding chewiness (Table 5), the values of this parameter increased as the degree of fat replacement increased. Thus, the CT sample showed the lowest values (*p* < 0.05), whereas the S50 sample exhibited the highest chewiness values (*p* < 0.05). The progressive increase in chewiness observed with increasing fat replacement may be associated with the structural changes induced by the incorporation of the vegetable-oil-based gelled emulsion. Unlike conventional animal fat, the gelled emulsion provides a structured lipid phase that may contribute to a more cohesive and mechanically resistant meat matrix [55].

### 3.5. Lipid Oxidation

**Figure 3:**
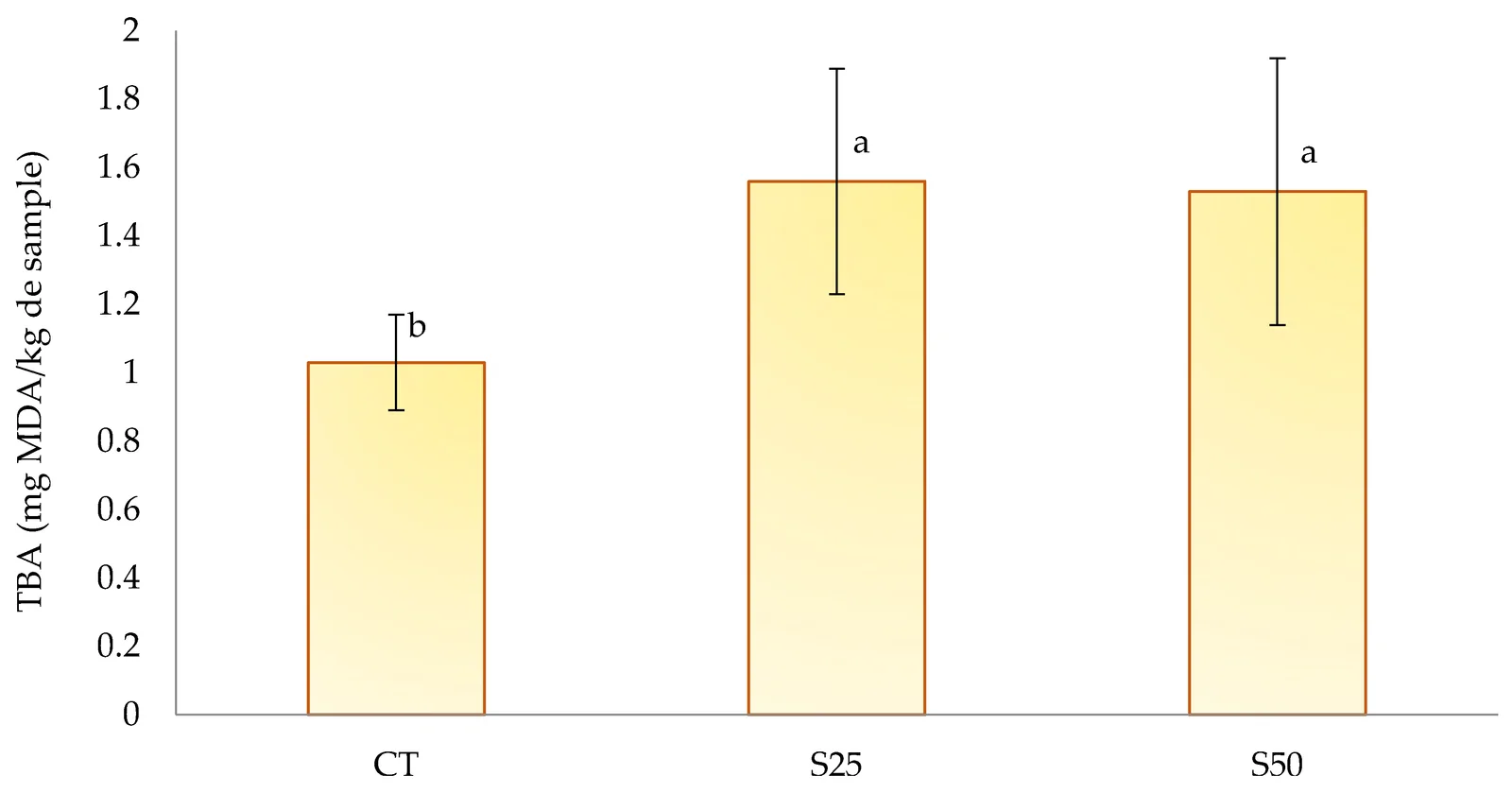
Lipid oxidation values of control “Longaniza de Pascua”-type sausage (CT) and “Longaniza de Pascua”-type sausage reformulated with a quinoa and walnut oil and pomegranate peel gelled emulsion used as a partial animal fat replacer. QWP-GE: oil-in-water emulsion prepared with quinoa and walnut oils and stabilized using pomegranate peel flour. CT: control sample produced with conventional fat content; S25: reformulated samples in which 25% of pork backfat was replaced by QWP-GE; S50: reformulated samples in which 50% of pork backfat was replaced by QWP-GE. Histograms sharing a superscript letter are not significantly different (*p* > 0.05), as determined by Tukey’s multiple range test.

Lipid oxidation is a key pathway of quality loss in dry-cured meat products, yielding secondary reaction products that impair flavor, generate off-odors, and degrade nutritional value [56]. Figure 3 shows the lipid oxidation values of control “Longaniza de Pascua”-type sausage and “Longaniza de Pascua”-type sausages reformulated with QWP-GE used as a partial animal fat replacer.

The control sample shows the lowest (*p* < 0.05) lipid oxidation values (1.03 mg MDA/kg sample), while for the S25 and S50 samples (1.56 and 1.53 mg MDA/kg sample, respectively), no differences (*p* > 0.05) were observed, indicating that the substitution of pork backfat with polyunsaturated oil emulsion increases susceptibility to oxidative degradation due to increases in the proportion of unsaturated fatty acids, which contain multiple double bonds and are highly susceptible to free-radical attack and peroxidation [57]. Although the incorporation of QWP-GE increased lipid oxidation in “Longaniza de Pascua”-type sausages, it is known that the acceptable limit of the oxidation degree is lower than 2 mg MDA/kg [58], a value that is associated with the threshold of consumer detection of rancid flavor. However, this value should be regarded as an indicative threshold rather than a universal limit, as the detection of rancidity depends on several factors, including the type of raw-cured meat product, its fat content, and individual consumer sensitivity as well as the analytical method used [59]. Increased lipid oxidation has been widely reported in meat products in which animal fat has been replaced with gelled emulsions containing vegetable oils. This fact occurs in fresh and cooked meat products [25,45] as well as in fermented and dry-cured products. For instance, Utrilla et al. [60] reported that *salchichón*, a dry-cured meat product, in which pork fat was replaced at different levels with an organogel formulated from olive oil, soy protein, and water, exhibited higher TBARS values (1.34–1.52 mg MDA/kg sample) than the control (1.13 mg MDA/kg sample). Similarly, Glisic et al. [61] found that replacing 16% of the fat in dry-fermented sausages with a gelled emulsion based on linseed oil and inulin resulted in greater lipid oxidation (1.25 mg MDA/kg sample) than in the control (0.85 mg MDA/kg sample) after one month of storage. Consistent with these findings, Öztürk-Kerimoğlu et al. [62] observed that fermented beef sausages formulated with peanut and linseed oils as fat replacers exhibited greater lipid oxidation than the corresponding control formulation.

### 3.6. Microbiological Properties

**Figure 4:**
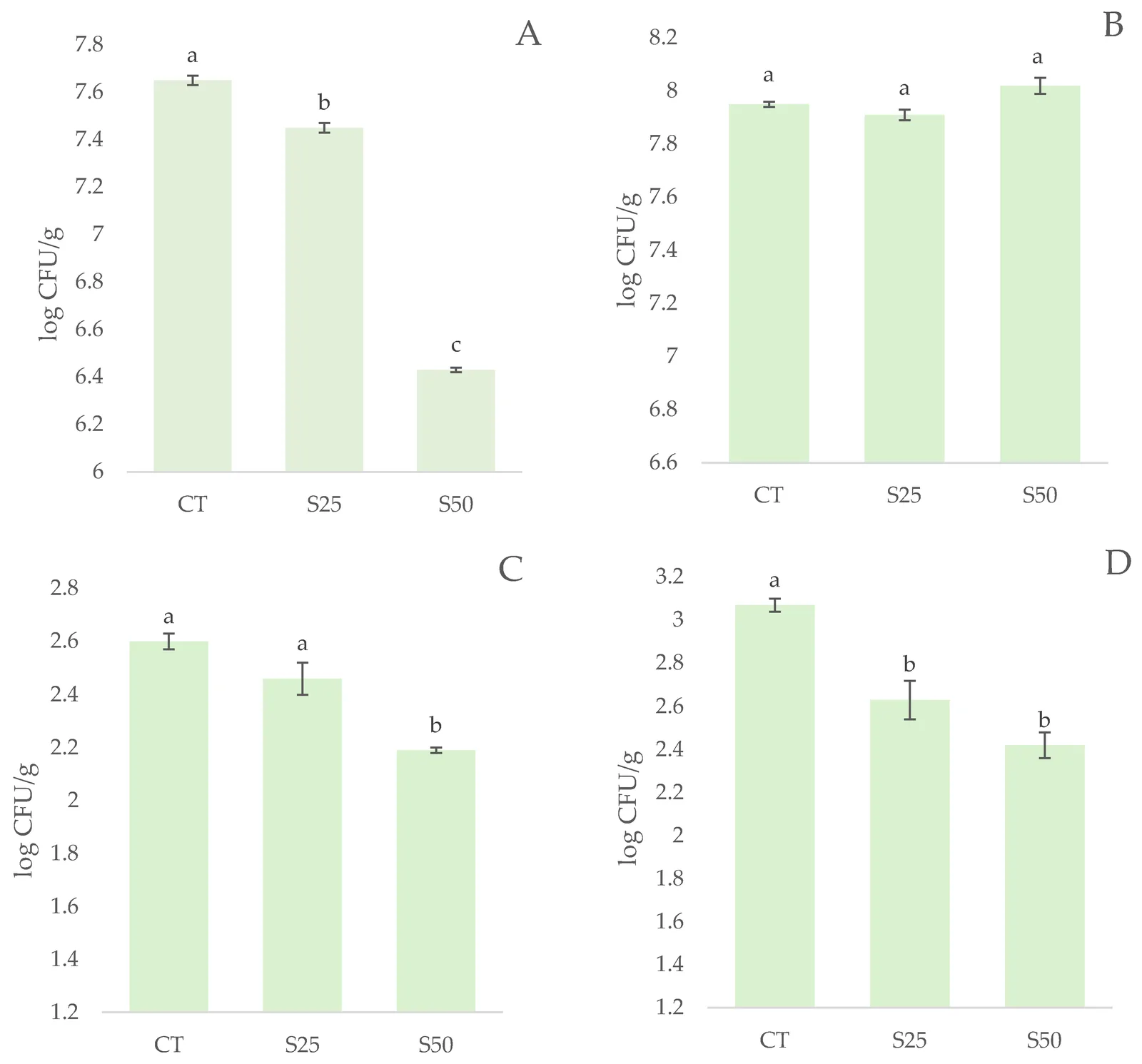
Microbiological analysis of control “Longaniza de Pascua”-type sausage (CT) and “Longaniza de Pascua”-type sausage reformulated with a quinoa/walnut oil and pomegranate peel gelled emulsion used as a partial animal fat replacer. (**A**): Aerobic mesophilic bacteria; (**B**): lactic acid bacteria; (**C**): molds; (**D**): yeast. QWP-GE: oil-in-water emulsion prepared with quinoa and walnut oils and stabilized using pomegranate peel flour. CT: control sample produced with conventional fat content; S25: reformulated samples in which 25% of pork backfat was replaced by QWP-GE; S50: reformulated samples in which 50% of pork backfat was replaced by QWP-GE. For each microbial strain (aerobic mesophilic bacteria, lactic acid bacteria, molds and yeast), histograms sharing a superscript letter are not significantly different (*p* > 0.05), as determined by Tukey’s multiple range test.

The results for microbiological analysis of the control “Longaniza de Pascua”-type sausage and “Longaniza de Pascua”-type sausages reformulated with QWP-GE utilized as a partial pork backfat replacer are given in Figure 4. For total aerobic counts (Figure 4A), the control sample showed the highest (*p* < 0.05) values (7.65 log CFU/g), followed by S25 (7.45 log CFU/g), while S50 had the lowest (*p* < 0.05) values. In the case of lactic acid bacteria (4B), no statistical differences (*p* > 0.05) were found between the control sample and the reformulated sausages in which QWP-GE gelled emulsion was used as a partial animal fat replacer. This suggests that the emulsion is compatible with LAB and does not inhibit fermentative microbiota. Regarding molds counts, no significant statistical differences were found (*p* > 0.05) between CT and S25, while S50 showed the lowest (*p* < 0.05) values (2.19 log CFU/g). On the other hand, in the case of yeast, CT showed the highest values (3.07 log CFU/g), while no significant statistical differences were found (*p* > 0.05) between S25 and S50. The microbiological values obtained for the control sample agree with those reported by Sayas-Barbera et al. [22] for this type of dry-cured meat product.

In the present study, the samples in which pomegranate peel flour was incorporate, as part of the gelled emulsion showed an effective reduction in the populations of aerobic bacteria, yeasts, and molds. In the scientific literature, it is well documented that pomegranate peel flour is a rich source of polyphenols, tannins, and ellagitannins, many of which exhibit antimicrobial activity against a broad range of spoilage microorganisms [63]. However, this activity appears to be highly selective. Thus, the lactic acid bacteria (LAB) were not significantly affected by any of the treatments. This differential response suggests a greater tolerance of LAB to the antimicrobial compounds present in pomegranate-derived ingredients.

One possible explanation for this resistance may be the metabolic capacity of certain LAB strains to enzymatically transform ellagitannins into lower-molecular-weight metabolites with reduced antimicrobial activity. Caballero et al. [64], for instance, demonstrated that selected strains belonging to five LAB species were capable of metabolizing punicalagin to ellagic acid. Although ellagic acid has been reported to exert antimicrobial activity against several pathogenic microorganisms [65], certain LAB may tolerate or metabolically accommodate this compound more effectively. Such metabolic adaptation could contribute to the detoxification of the local environment and reduce the inhibitory effects of pomegranate-derived polyphenols on LAB. This mechanism may, at least in part, explain the differential susceptibility observed between LAB and other microbial groups in the present study. It is very important to highlight that in both “Longaniza de Pascua”-type sausage and “Longaniza de Pascua”-type sausages reformulated with QWP-GE, *Enterobacteriaceae* strains were not found, showing that the “Longaniza de Pascua” elaboration process was conducted under strictly controlled hygienic conditions when using raw materials (pork lean, pork backfat and QWP-GE), without contamination by these types of microorganisms. Additionally, pH and water activity values combined with competitive microflora could have affected *Enterobacteriaceae* growth, as occurs in the work carried out by Siachou et al. [36], with the use of bigels as fat replacers in fermented sausages.

## 4. Conclusions

The partial replacement of pork backfat with a quinoa- and walnut-oil-based gelled emulsion containing pomegranate peel flour (QWP-GE) highlighted a clear trade-off between nutritional improvement and technological performance in “Longaniza de Pascua”-type sausage. On the nutritional side, reformulation reduced the fat content and improved the fatty acid profile, particularly by decreasing the proportion of saturated fatty acids and increasing the contribution of unsaturated fatty acids, resulting in more favorable calculated lipid nutritional indices. However, these benefits were accompanied by increased hardness, changes in color attributes and greater susceptibility to lipid oxidation, likely associated with the higher proportion of polyunsaturated fatty acids introduced through the vegetable oils. Therefore, the main finding is that improving the nutritional profile of this traditional dry-cured sausage through fat reformulation involves balancing the benefits of reduced fat content and improved lipid quality against potential drawbacks in texture, appearance and oxidative stability.

Despite the increased susceptibility to lipid oxidation, the oxidation levels measured in the reformulated sausages remained within the range considered acceptable according to the criterion discussed in this study. Nevertheless, this finding does not eliminate the need for the further investigation of oxidative stability during processing and storage. The reformulation did not adversely affect the development of lactic acid bacteria, and Enterobacteriaceae were not detected in the finished products under the conditions investigated. These microbiological results are encouraging but do not, by themselves, establish the microbiological safety of the product throughout manufacture and storage.

These findings show that the use of quinoa and walnut oil-based gelled emulsions containing pomegranate peel flour represents a promising strategy for modifying the lipid composition and reducing the fat content of traditional dry-cured sausages, but its application requires careful consideration of the associated technological changes. Further research should focus on optimizing texture and color, improving oxidative stability, assessing sensory acceptability and validating the formulation under pilot- or industrial-scale processing and storage conditions before commercial implementation.

## Figures and Tables

**Table 1:** Formulation of control “Longaniza de Pascua”-type sausage (CT) and “Longaniza de Pascua”-type sausage reformulated with QWP-GE used as a partial animal fat replacer at 25% (S25) and 50% (S50).

	Control	S25	S50
Pork lean meat	53.58	53.58	53.58
Pork backfat	35.73	26.80	17.86
QWP-GE	0	8.93	17.86
Ice	8.93	8.93	8.93
NaCl	1.34	1.34	1.34
Ascorbic acid	0.05	0.05	0.05
Nitrite	0.012	0.012	0.012
Garlic powder	0.09	0.09	0.09
Black pepper	0.18	0.18	0.18
Anise	0.09	0.09	0.09

Values expressed as g/100 g. QWP-GE: oil-in-water emulsion prepared with quinoa and walnut oils and stabilized using pomegranate peel flour. The 25% and 50% replacement levels refer to the mass of pork backfat replaced on a 1:1 mass basis with QWP-GE rather than to an equivalent amount of lipid.

**Table 2:** Chemical composition of control “Longaniza de Pascua”-type sausage (CT) and “Longaniza de Pascua”-type sausage reformulated with QWP-GE used as a partial animal fat replacer at 25% (S25) and 50% (S50).

	Moisture	Protein	Fat	Ash	NaNO_2_
CT	31.66 ± 0.36 ^a^	33.70 ± 0.26 ^c^	51.52 ± 0.68 ^a^	5.60 ± 0.14 ^b^	1.06 ± 0.12 ^a^
S25	30.90 ± 0.13 ^a^	37.18 ± 0.19 ^b^	45.87 ± 0.32 ^b^	6.39 ± 0.13 ^ba^	0.71 ± 0.14 ^a^
S50	25.07 ± 0.57 ^b^	38.19 ± 0.38 ^a^	41.03 ± 1.16 ^c^	6.19 ± 0.16 ^a^	0.23 ± 0.01 ^b^

Values expressed as mean ± standard deviation of three independent replicates (n = 3). Moisture values expressed as g/100 g; protein, fat and ash values expressed as g/100 g dry basis; NaNO_2_ values expressed as mg/kg sample. QWP-GE: oil-in-water emulsion prepared with quinoa and walnut oils and stabilized using pomegranate peel flour. CT: control sample produced with conventional fat content; S25: reformulated samples in which 25% of pork backfat was replaced by QWP-GE; S50: reformulated samples in which 50% of pork backfat was replaced by QWP-GE. Within each column, values sharing a superscript letter are not significantly different (*p* > 0.05), as determined by Tukey’s multiple range test.

**Table 3:** Fatty acid profile of control “Longaniza de Pascua”-type sausage (CT) and “Longaniza de Pascua”-type sausage reformulated with QWP-GE used as a partial animal fat replacer at 25% (S25) and 50% (S50).

	CT	S25	S50
	Saturated Fatty Acids (SFAs)		
C10:0	0.08 ± 0.00 ^a^	0.07 ± 0.00 ^b^	0.06 ± 0.00 ^c^
C12:0	0.13 ± 0.00 ^a^	0.12 ± 0.00 ^a^	0.12 ± 0.00 ^a^
C14:0	1.42 ± 0.02 ^a^	1.27 ± 0.03 ^b^	1.07 ± 0.04 ^c^
C15:0	0.05 ± 0.00 ^a^	0.04 ± 0.00 ^b^	0.04 ± 0.00 ^b^
C16:0	24.61 ± 0.09 ^a^	22.21 ± 0.20 ^b^	19.41 ± 0.43 ^c^
C17:0	0.25 ± 0.01 ^a^	0.24 ± 0.01 ^a^	0.24 ± 0.02 ^a^
C18:0	14.03 ± 0.08 ^a^	12.56 ± 0.05 ^b^	10.51 ± 0.16 ^c^
C20:0	0.99 ± 0.02 ^a^	0.82 ± 0.00 ^b^	0.65 ± 0.01 ^c^
C23:0	0.14 ± 0.00 ^a^	0.14 ± 0.01 ^a^	0.14 ± 0.00 ^a^
	Unsaturated Fatty Acids (UFAs)		
C16:1 n-9	2.15 ± 0.02 ^a^	1.83 ± 0.02 ^b^	1.46 ± 0.05 ^c^
C17:1 n-9	0.21 ± 0.00 ^a^	0.19 ± 0.00 ^b^	0.16 ± 0.00 ^c^
C18:1 n-9	42.19 ± 0.01 ^a^	38.38 ± 0.10 ^b^	34.06 ± 0.38 ^c^
C18:2 n-6	12.08 ± 0.05 ^c^	19.06 ± 0.26 ^b^	27.38 ± 0.88 ^a^
C18:3 n-3	0.65 ± 0.00 ^c^	2.00 ± 0.04 ^b^	3.66 ± 0.17 ^a^
C20:2 n-11	0.59 ± 0.00 ^a^	0.51 ± 0.00 ^b^	0.41 ± 0.01 ^c^
C20:3 n-8	0.40 ± 0.01 ^a^	0.38 ± 0.00 ^b^	0.33 ± 0.00 ^c^
C20:3 n-6	0.11 ± 0.02 ^a^	0.10 ± 0.10 ^a^	0.09 ± 0.01 ^a^
C20:2 n-6	0.00 ± 0.00 ^c^	0.07 ± 0.02 ^b^	0.12 ± 0.01 ^a^
∑ SFA	41.70 ± 0.13 ^a^	37.47 ± 0.27 ^b^	32.30 ± 0.65 ^c^
∑ MUFA	44.48 ± 0.10 ^a^	40.40 ± 0.10 ^b^	35.68 ± 0.43 ^c^
∑ PUFA	13.84 ± 0.06 ^c^	22.13 ± 0.28 ^b^	31.98 ± 1.04 ^a^
∑ UFA	58.32 ± 0.13 ^c^	62.53 ± 0.27 ^b^	67.66 ± 0.63 ^a^
∑ n3	0.65 ± 0.00 ^c^	2.07 ± 0.02 ^b^	3.78 ± 0.18 ^a^
∑ n6	12.2 ± 0.06 ^c^	19.16 ± 0.26 ^b^	27.47 ± 0.87 ^a^

Values expressed as g/100 g of fat as mean ± standard deviation of three independent replicates (n = 3). QWP-GE: oil-in-water emulsion prepared with quinoa and walnut oils and stabilized using pomegranate peel flour. CT: control sample produced with conventional fat content; S25: reformulated samples in which 25% of pork backfat was replaced by QWP-GE; S50: reformulated samples in which 50% of pork backfat was replaced by QWP-GE. Within each column, values sharing a superscript letter are not significantly different (*p* > 0.05), as determined by Tukey’s multiple range test.

**Table 4:** Health indices of control “Longaniza de Pascua”-type sausage (CT) and “Longaniza de Pascua”-type sausage reformulated with QWP-GE used as a partial animal fat replacer at 25% (S25) and 50% (S50).

	AI	TI	h/H	SFAs/UFAs	n3/n6
CT	0.52 ± 0.00 ^a^	1.32 ± 0.01 ^a^	2.14 ± 0.01 ^c^	0.71 ± 0.00 ^a^	0.05 ± 0.00 ^c^
S25	0.44 ± 0.01 ^b^	1.00 ± 0.01 ^b^	2.57 ± 0.04 ^b^	0.60 ± 0.01 ^b^	0.11 ± 0.00 ^b^
S50	0.35 ± 0.01 ^c^	0.72 ± 0.03 ^c^	3.20 ± 0.10 ^a^	0.48 ± 0.01 ^c^	0.14 ± 0.00 ^a^

Values expressed as mean ± standard deviation of three independent replicates (n = 3). QWP-GE: oil-in-water emulsion prepared with quinoa and walnut oils and stabilized using pomegranate peel flour. CT: control sample produced with conventional fat content; S25: reformulated samples in which 25% of pork backfat was replaced by QWP-GE; S50: reformulated samples in which 50% of pork backfat was replaced by QWP-GE. SFAs: saturated fatty acids. UFAs: unsaturated fatty acids. n-3: omega-3; n-6: omega-6; AI: atherogenic index; TI: thrombogenic index; h/H: hypo cholesterolemic/hypercholesterolemic index. Within each column, values sharing a superscript letter are not significantly different (*p* > 0.05), as determined by Tukey’s multiple range test.

**Table 5:** Physico-chemical properties of control “Longaniza de Pascua”-type sausage (CT) and “Longaniza de Pascua”-type sausage reformulated with QWP-GE used as a partial animal fat replacer at 25% (S25) and 50% (S50).

		CT	S25	S50
	pH	5.42 ± 0.05 ^a^	5.30 ± 0.04 ^b^	5.05 ± 0.05 ^c^
Color properties	L*	44.51 ± 1.67 ^a^	45.55 ± 2.37 ^a^	44.01 ± 1.66 ^a^
	a*	7.02 ± 0.59 ^a^	5.98 ± 0.61 ^b^	6.02 ± 0.48 ^b^
	b*	7.37 ± 1.49 ^b^	11.42 ± 1.87 ^a^	11.19 ± 1.14 ^a^
	C*	10.25 ± 0.86 ^b^	13.28 ± 1.02 ^a^	12.87 ± 1.13 ^a^
	h	48.15 ± 1.85 ^b^	58.83 ± 1.92 ^a^	60.70 ± 1.82 ^a^
	∆E	---	8.37 ± 0.93 ^a^	7.46 ± 0.06 ^b^
Textural properties	Hardness (N)	106.17 ± 2.10 ^b^	117.90 ± 8.64 ^a^	128.76 ± 2.10 ^a^
	Springiness (mm)	0.42 ± 0.04 ^a^	0.41 ± 0.07 ^a^	0.45 ± 0.07 ^a^
	Cohesiveness	0.27 ± 0.03 ^b^	0.33 ± 0.02 ^a^	0.31 ± 0.02 ^ab^
	Gumminess (N)	28.40 ± 1.75 ^b^	38.59 ± 1.50 ^a^	40.20 ± 1.04 ^a^
	Chewiness (N·mm)	11.97 ± 0.47 ^c^	16.06 ± 0.74 ^b^	18.16 ± 0.51 ^a^

Values expressed as mean ± standard deviation of three independent replicates (n = 3). QWP-GE: oil-in-water emulsion prepared with quinoa and walnut oils and stabilized using pomegranate peel flour. CT: control sample produced with conventional fat content; S25: reformulated samples in which 25% of pork backfat was replaced by QWP-GE; S50: reformulated samples in which 50% of pork backfat was replaced by QWP-GE. Within each row, values sharing a superscript letter are not significantly different (*p* > 0.05), as determined by Tukey’s multiple range test.

## Data Availability

Data will be made available on request.

## Supplementary Materials

The following supporting information can be downloaded at https://www.mdpi.com/article/10.3390/foods15214001/s1, Table S1: Chemical composition expressed on a fresh-weight basis of control “Longaniza de Pascua”-type sausage (CT) and “Longaniza de Pascua”-type sausage reformulated with QWP-GE used as a partial animal fat replacer at 25% (S25) and 50% (S50).
