# Supplementary material for: Assessment of Walnut and Quinoa Oil-Based Gelled Emulsion as Partial Animal Fat Replacer in “Longaniza de Pascua” Sausage, a Dry-Cured Meat Product
Source: Foods. 2026 Nov 7;15(21):4001. doi: 10.3390/foods15214001 (PMC13763416; doi:10.3390/foods15214001)
Supplement: Supplementary file 1 [file foods-15-04001-s001.zip › foods-4618554-supplementary.pdf]

Supplementary Material

Supplementary Table S1. Chemical composition expressed in fresh-weight basis of control "Longaniza de Pascua"-type sausage (CT) and "Longaniza de Pascua"-type sausage reformulated with QWP-GE used as partial animal fat replacers at 25% (S25) and 50% (S50).

	**Moinsture**	**Fat**	**Protein**	**Ash**
CT	31.66±0.36	35.21±0.68	25.25±0.26	3.83±0.07
S25	30.90±0.13	33.08±0.32	25.41±0.19	4.42±0.01
S50	25.07±0.57	33.74±1.16	26.39±1.18	4.64±0.11

Values expressed as g/100g
